# Effectiveness of autodissemination stations containing pyriproxyfen in reducing immature *Aedes albopictus* populations

**DOI:** 10.1186/s13071-017-2034-7

**Published:** 2017-03-09

**Authors:** Isik Unlu, Devi S. Suman, Yi Wang, Kim Klingler, Ary Faraji, Randy Gaugler

**Affiliations:** 1 Center for Vector Biology, Rutgers University, 180 Jones Avenue, New Brunswick, NJ 08901 USA; 2 Mercer County Mosquito Control, 300 Scotch Road, West Trenton, NJ 08628 USA; 3 Salt Lake City Mosquito Abatement District, Salt Lake City, UT 84116 USA

**Keywords:** Asian tiger mosquito, Autodissemination station, Pyriproxyfen, Egg surveillance, Adult surveillance

## Abstract

**Background:**

*Aedes albopictus*, the Asian tiger mosquito, is an aggressive, highly anthropophilic, day-biting mosquito with an expanding geographic range. Suppression of *Ae. albopictus* is difficult because of the abundance and prevalence of larval habitats within peridomestic environments, particularly cryptic habitats such as corrugated extension spouts, fence post openings, discarded food containers, etc. Because of the challenges of eliminating or treating larval habitats of this species, we tested an autodissemination concept to contaminate these habitats with the insect growth regulator pyriproxyfen.

**Methods:**

Our study was conducted in the City of Trenton (Mercer County), New Jersey, USA (40°12′N, 74°44′W). We selected six hot spots, where five or more *Ae. albopictus* males or females were collected based on weekly trap surveillance. A trapping unit was a city block, approximately 0.8 ha (hot spot), where we deployed 26 to 28 autodissemination stations per treatment plot. To gauge efficacy, we deployed BGS traps, oviposition cups, and sentinel cups in treatment and control locations.

**Results:**

We found a significant reduction in eggs (*P* < 0.0001) and larval populations (*P* < 0.0001) as a result of treatment. Pupal mortality, as determined through bioassays, was also significantly higher in the treatment sites (*P* < 0.0001).

**Conclusion:**

Our results clearly show the potential and unique use of the autodissemination stations to control immature *Ae. albopictus* in urban areas. Penetration of larvicides with existing methods are difficult to reach cryptic habitats, but the autodissemination approach, which exploits the oviposition behavior of the target pest, can be integrated into intervention programs. New tools are urgently needed to curb the expansion and public health implications of *Ae. albopictus* and other container-inhabiting species.

**Electronic supplementary material:**

The online version of this article (doi:10.1186/s13071-017-2034-7) contains supplementary material, which is available to authorized users.

## Background

Recent outbreaks of chikungunya (CHIKV) and Zika (ZIKV) virus infections have stimulated increased interest in management strategies for vector species such as *Aedes aegypti* (L.) and *Aedes albopictus* (Skuse) [1–4]. The latter species has been reported as the sole vector of CHIKV in explosive epidemics on the islands of La Réunion and Mauritius [2], and a potential secondary vector of ZIKV [4]. The CHIKV outbreak was caused by a new variant of the virus [Reunion Island CHIKV isolates (CHIKV 226OPY1)] with a single adaptive mutation, an amino acid change from alanine to valine in the E1 glycoprotein which increased the infection and dissemination of the virus in *Ae. albopictus* [5–7]. Since there are no vaccines or specific antiviral treatments available for CHIKV or ZIKV, *Ae. albopictus* surveillance and control is critical for the prevention of outbreaks.

*Aedes albopictus* is a container-inhabiting mosquito, which in its native range of southeastern and eastern Asia, oviposits in tree holes, bamboo nodes, and a variety of artificial containers [8]. The adaptation of this species to exploit artificial containers, in combination with its affinity for urban environments where containers such as buckets and tires are abundant, poses a challenge to control this species [9–12]. *Aedes albopictus* also exhibits skip oviposition, where the species may oviposit eggs from the same batch in multiple containers [13, 14]. Most water-holding containers within the peridomestic environment are suitable larval habitats for *Ae. albopictus* [9, 15]. Previous studies have reported that corrugated extension spouts routinely contain larvae and pupae of *Ae. albopictus* in residential backyards of urban and suburban areas of northeastern USA [16].

Suppressing populations of *Aedes albopictus* has been traditionally more difficult than *Ae. aegypti*, in part because of differences in their oviposition preferences [17, 18]. *Aedes albopictus* prefer numerous smaller habitats, which is much different than wetland mosquito species that develop in habitats that are large and predictable [9]. We have also shown in previous studies [16] that immature *Ae. albopictus* numbers were higher in cryptic corrugated extension spouts (containers positioned on the ground horizontally) than open containers (containers positioned on the ground vertically) during the peak season (August). Because of the affinity of *Ae. albopictus* for cryptic habitats, area-wide larviciding using backpacks [19] or truck-mounted sprayers are not optimal control measures due to the poor penetration of pesticides into these habitats [16, 20]. Door-to-door source reduction campaigns are also labor and time intensive, often leading to ineffective control outcomes [21–23]. The autodissemination approach may be a means of insecticide delivery into cryptic habitats that are otherwise hard to treat by conventional methods.

Autodissemination is a ‘pull’ (attraction and adhesion) and ‘push’ (dispersal and transfer) technology which allows mosquito control professionals to treat larval habitats in a timely and economical fashion. Briefly, autodissemination is a pest management method in which insects contaminated with an insecticide, transfer lethal concentrations horizontally or vertically to other insects via mating, oviposition, aggregation and other behaviors [24–27]. The success of autodissemination stations depends on three criteria: (i) attraction of mosquitoes to the stations; (ii) transfer of chemicals to the mosquitoes; and (iii) dissemination of chemicals to target habitats [24]. Gaugler et al. [24] achieved all three conditions with their 2012 design during semi-field trials and successfully contaminated containers using mosquitoes as the vehicles. A novel ovitrap was developed based on pull and push strategy to control *Ae. aegypti* by Snetselar et al. [28], using a combination of attractant media, *Beauveria bassina* (an entomopathogenic fungi), and pyriproxyfen (a biopesticide). Researchers achieved 100% larval mortality in the laboratory; however, the study did not include any field trials. The most commonly used chemical in autodissemination stations has been the insect growth regulator (IGR) pyriproxyfen, which does not cause immediate mortality or impair adult activity [29], but is effective on immature mosquitoes at extraordinarily low concentrations (LC_50_ in *Ae. albopictus* 0.012 ppb and *Ae. aegypti* is 0.023 ppb) [24, 30]. This approach seems to be a feasible strategy based on *Ae. albopictus* and *Ae. aegypti* skip-oviposition behavior, facilitated by distributing eggs from a single gonothrophic cycle [31]. Devine at al. [32] achieved 42–98% *Ae. aegypti* adult emergence inhibition by using pyriproxyfen-treated stations in Peru [32]. Autodissemination has also been tested by different groups in small scale field experiments against two dengue vectors [10, 26, 32]. Recently, Abad-Franch et al. [33] evaluated the efficacy of emulsifiable pyriproxyfen stations in a tropical neighborhood by monitoring *Ae. aegypti*, *Ae. albopictus* and *Culex* spp. populations. This approach resulted in a 10-fold decrease in adult mosquito emergence. However, large scale autodissemination applications need further investigation. A novel approach, “Auto-Dissemination Augmented by Males” (ADAM), has proven that pyriproxyfen-dusted males were able to transmit lethal amounts of insecticide to oviposition sites, resulting in reduced adult populations of *Ae. albopictus* [26]. Most studies have primarily shown reduction of immature and adult populations caused by pupal mortality, and reduced egg number of *Ae. aegypti* females exposed to sub-lethal doses of pyriproxyfen [34, 35]. In the current study, we evaluated the effects of deployed autodissemination stations in *Ae. albopictus* hot spots (localized areas with high numbers of adults) through monitoring of eggs, larvae, pupae and adult populations in a temperate urban area, Trenton, New Jersey, USA.

## Methods

### Study site description

Our study was conducted in the City of Trenton (Mercer County), New Jersey, USA (40°12′N, 74°44′W). Trenton sites were urban residential neighborhoods: South Olden (40°22′N, 74°73′W) was 48.6 ha consisting of 24 city blocks, each containing a residential street on all four sides and divided lengthwise by a drivable alley. South Olden consisted of 1250 parcels (i.e. house with surrounding yard), most often built as adjoining row homes or duplexes [36]. Parcel sizes were approximately 200 m^2^. We have continuous *Ae. albopictus* surveillance data between 2008 and 2014 from this study site, demonstrating high *Ae. albopictus* populations [9, 22, 37].

### Identification of *Ae. albopictus* hot spots

**Fig. 1:**
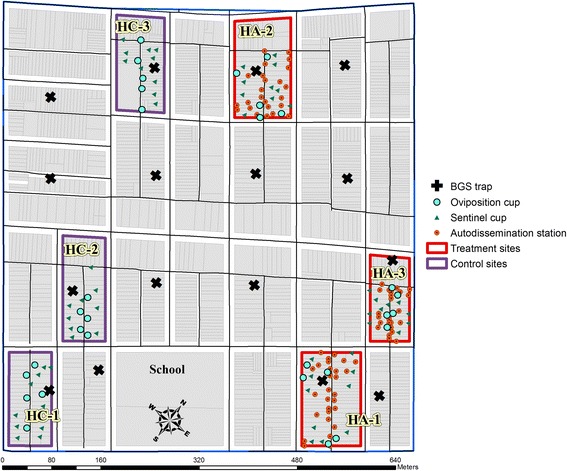
Map of the study locations, including trapping sites (*n* =1), and the locations of oviposition cups (*n* =5), sentinel cups (*n* =10), and autodissemination stations (26–28) in treatment and control sites, Trenton, New Jersey, 2014

We monitored adult populations of *Ae. albopictus* with BG Sentinel^TM^ traps (BGS traps; Biogents AG, Regensburg, Germany). A trapping site was a city block with a BGS trap deployed in a parcel located in the center (or as close as to the middle location as possible). A trapping site was classified as a hot spot when five or more *Ae. albopictus* males and females were collected in that individual trapping site based on weekly trapping surveillance [36]. This number was chosen because three bites have been reported as a common nuisance threshold driving residents indoors, and an average of five bites per day by *Ae. albopictus* has been recorded as intolerable [38–41]. For this study we used females and males for the nuisance threshold determination because *Ae. albopictus* is very focal within our study sites and male numbers are often followed by increasing female numbers in the traps [37]. We identified 6–10 hot spots based on 16 trapping sites between June 13 and July 23, 2014. Trapping sites that were identified as hot spots at least three times were selected for this study. To reduce the chances of control contamination, the site was split in two parts (treatment versus control) using the center most road as a dividing line (Fig. 1). Hot spots were often determined by the boundaries of a city block (Fig. 1), approximately 0.8 ha where we deployed 26 to 28 autodissemination stations per treatment site. Treatment sites are hereafter referred to as HA1 (hot spot autodissemination 1), HA2 and HA3, and control sites as HC1 (hot spot control 1), HC2 and HC3 (Fig. 1).

To reduce the immigration from outside the study sites, we conducted area-wide larviciding with a water-dispersible granular (WDG) formulation of *Bacillus thuringienis* var. *israelensis* (Bti; VectoBac® WDG, Valent BioSciences Corp., Libertyville, IL, USA) prior to autodissemination station deployment. This was conducted within the barrier zone created around the sites, but excluded the experimental sites. We used a CSM2 Mist Sprayer (Buffalo Turbine, Springville, NY, USA) [20], for the Bti applications, and conducted the first application on 27 July between 01:00 and 06:00 h. Following the initial larviciding, ultra-low volume (ULV) adulticide applications using DUET^TM^ Dual-action Adulticide (Clarke Mosquito Control, Roselle, IL, USA) were conducted each week around the treatment and control sites until 26 September. A Cougar® (Clarke Mosquito Control, Roselle, IL, USA) cold aerosol ULV generator was used during all adulticide applications. Truck-mounted adulticide applications were conducted at night using a single vehicle to drive buffer areas including all available roads and alleys to provide maximum coverage. Each application took about 2 h to complete and was conducted between 01:00 and 4:00 h [37].

### Autodissemination stations

**Fig. 2:**
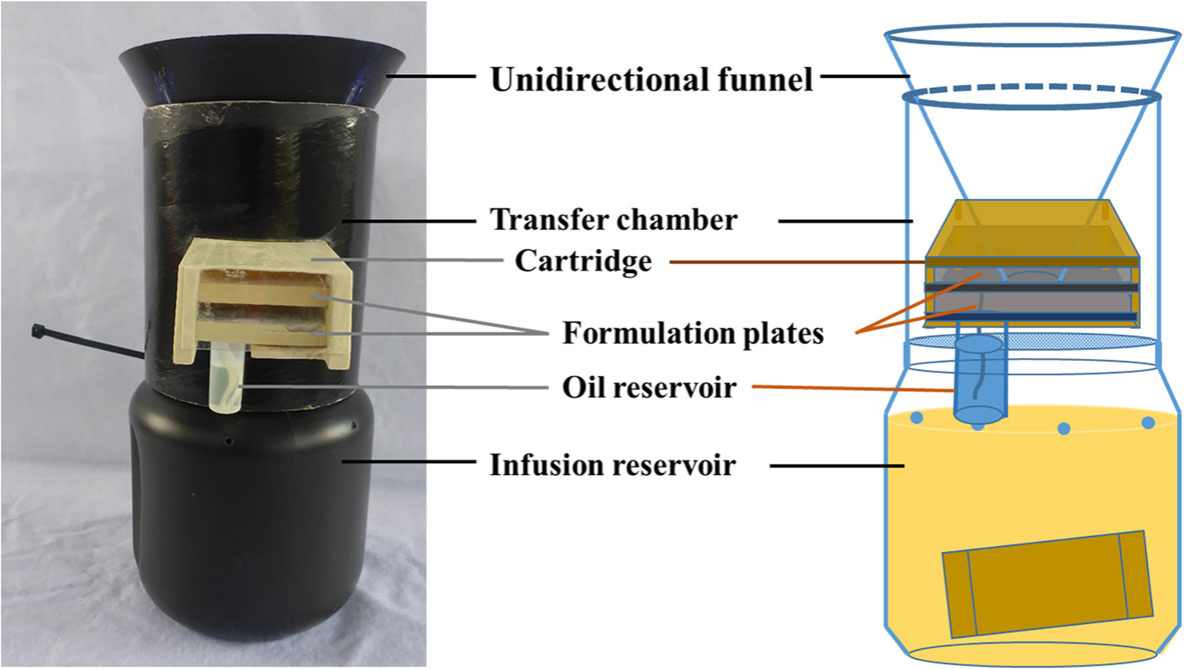
Autodissemination stations consisting of an infusion reservoir, transfer chamber, and a unidirectional funnel

The autodissemination stations were modified from an earlier design [24] by introducing the mechanism of dual treatment [25]. The station consisted of a transfer chamber, unidirectional funnel, and an infusion reservoir (Fig. 2). The transfer plate used by Wang et al. [25] was modified into a cartridge, which was inserted into the middle of the chamber body. The cartridge contains two formulation plates (top and bottom) and each plate (55 × 50 mm) contains an oil and powder band. The oil bands were coated with an oil formulation containing 20% pyriproxyfen a.i. (0.62 g/station) and the powder bands were coated with a powder formulation containing 60% pyriproxyfen a.i. (0.42 g/station). The oil formulation was coated by wicking the oil from a reservoir located on the bottom of the cartridge, which ensures availability of the oil formulation over the entire active season. The space for exiting gravid mosquitoes was 6 mm, which forced them to contact the oil formulation before picking up the powder. Gravid mosquitoes attracted by the infusion in the reservoir enter from the top of the unidirectional funnel to attempt to lay eggs. However, the mesh on top of the infusion reservoir prevents them from reaching the infusion. Fail to find a suitable oviposition site, the gravid mosquitoes are forced to exit the station from the gaps between formulation plates where they get contaminated with the oil formulation first and then pick up the powder formulation before exiting the station. The formulation is then transferred into the containers where the contaminated mosquitoes seek oviposition. Additionally, the earlier station used oak leaf infusion [22], whereas here oak leaves were packed in mesh bags and placed into the reservoir water in an effort to extend attraction and duration. The tea-bag containing 7.5 g of shredded oak leaves and 15 g of oak wood chips was preloaded in the reservoir. The station was tested in a room assay (30 m^3^) using 10 oviposition cups filled with 250 ml of water by releasing 50 gravid females. The autodissemination station consistently achieved 100% pupal mortality within all oviposition cups. Oviposition cups were placed in the control and treatment sites on 28 July 2014 and autodissemination stations were deployed in the treatment sites only. Autodissemination stations were serviced weekly to ensure proper function, and field crews unblocked the opening of clogged stations which may have been caused by leaves or spider webs.

### Egg surveillance

Black 360 ml oviposition cups (SpringStar, Inc., Woodinville, WA, USA)] were zip-tied to a fence at ground level to reduce disturbance. The oviposition cups were filled with 300 ml of tap water, and seed germination paper was used to cover the inside surface acting as oviposition substrate. Two small holes were pre-drilled above the water line to prevent them from overflowing after a rain event. We deployed 30 oviposition cups (5 × 3 treatment sites, 5 × 3 control sites, Fig. 1). The oviposition papers were changed at 3 to 5 day intervals to coincide with adult trapping. Positive egg papers were subsampled (5 egg papers per week) and submersed in dechlorinated water in the laboratory. Because *Aedes japonicus japonicus* (Theobald) and *Aedes triseriatus* (Say) eggs other two mosquito species commonly found in containers in New Jersey [42], are very similar to *Ae. albopictus* eggs, larval food was provided until immatures reached third-instar stage and identified. Over 98% of the larvae were identified as *Ae. albopictus* [43].

### Larval surveillance and pupal mortality

Initially, 10 sentinel cups were placed at each hot spot and filled with 250 ml of dechlorinated tap water (Fig. 1). To improve stability during inclement weather or other disturbances, we developed an anchoring system using a similar black cup with a hole in the bottom where a 20 cm galvanized spike could be driven securely into the ground, creating a nest to hold the sentinel cups in place. The sentinel cups were sampled at 2 week intervals as fresh water are less preferred in comparison to natural habitats [44]. Longer exposure provides the opportunity to deliver sufficient pyriproxyfen into cups through multiple visits of mosquitoes, which compensates for absorption of pyriproxyfen in sentinel cups. The original cups were left in the field for the entirety of the study. If a collected cup was below 250 ml or empty, additional dechlorinated water was added as needed, agitated in the cup, and poured into the new sample cup for bioassays. After sampling, the sentinel cups were replenished with 250 ml of water and returned to their corresponding locations. The control sites were always sampled first and placed in an enclosed container inside the cab of the vehicle, while the treatment samples were placed in the back of the vehicle upon completion. Gloves were changed for each sampling to prevent contamination. Each sentinel cup from the control and treatment hot spots were observed, and the number of larvae, pupae and pupal cases in each container was recorded. After counting immature mosquito populations, sentinel cups were filtered to remove debris, organic materials and immature mosquitoes. A 50 ml water sample was taken from the sentinel cups for pyriproxyfen residue analysis. The residue analysis of pyriproxyfen was carried out at Golden Pacific Laboratories (CA, USA) using a liquid-mass-mass spectrophotometry (LC-MS-MS) analysis as described by Wang et al. [25]. For the analysis, sample thawed, diluted 1:1 with acetonitrile and aliquot was passed through 0.45 μm filters. Primary and secondary ion pairs was monitored for the analysis using an AB Sciex API 5000 mass spectrophotometer. Each set of samples contained water blank and positive controls fortified at the limit of quantification and at a higher level to bracket expected sample concentrations. With the remaining 200 ml, we conducted larval bioassay by inoculating 20 third instars at 26 ± 1 °C and 16:8 L:D photoperiod. For colony mosquitoes, larval food (brewer’s yeast, 30 mg/l) was provided twice a week. Larval and pupal mortality and adult emergence were recorded to estimate efficacy. For negative control, three cups were set up using distilled water and 20 larvae per bioassay. Mosquito colony information and maintenance are described in detail elsewhere [25]. Briefly, larvae obtained for bioassays were from an *Ae. albopictus* colony established from the eggs of field populations collected in Mercer County, New Jersey, USA between 2008 and 2010 [25]. Restrained guinea pigs were used to blood-fed the females (Rutgers University Animal Use Protocol No. 86–129) and eggs were collected on seed germination paper and stored at 26 ± 1 °C. Third stage larvae were used in all bioassays.

### Adult surveillance

We used BGS traps for adult mosquito sampling [37, 40], baited with BG lures (Biogents AG, Regensburg, Germany). Details of surveillance protocols are outlined elsewhere [22]; in short, trapping sites were chosen by overlaying a grid of 175 m intervals based on *Ae. albopictus* flight range [18] and available traps and personnel. The Fishnet tool within ArcGIS Desktop 9.2 (Environmental Systems Research Institute, Redlands, CA, USA) was used to determine potential trapping sites. The 175 m grid resulted in 16 traps within the study site (Fig. 1). Permission to place BGS traps was acquired from property owners. Traps were deployed in the field continuously for 24 h once per week during the mosquito season, and all mosquitoes were identified to species and gender [37]. Trapping started 14 May and ended 12 November when no mosquitoes were collected for 2 to 3 weeks.

### Data analysis

The effect of placement of autodissemination station in the treatment sites was investigated by comparing the number of eggs collected from oviposition cups at the treatment sites (HA 1-3) to the number of eggs collected from oviposition cups at the control sites (HC 1-3) using negative binomial regression (PROC GENMOD, SAS version 9.3; SAS Institute 2011), with treatment, week, and treatment*week as predictors, where treatment is an indicator variable (1 if site contained Autodissemination station, 0 otherwise). Since the number of days oviposition cups remained in the field varied, the natural log of the number of days a oviposition cup remained in the field was used as an offset. The number of larvae occurring in sentinel cups collected from the control and treatment sites were compared by negative binomial regression in SAS using the default log link. None of the sentinel cups contained larvae in the second week, therefore, data from this week were not included in the analysis. The model used treatment as a predictor for larval data showed signs of misfit, so instead the model was fitted using site, week, and site*week as predictors. Comparison of the mean number of larvae collected over the three treatment sites and three control sites was accomplished using a linear contrast. The mean pupal mortality from the laboratory bioassays using water samples collected from sentinel cups from all sites were compared by negative binomial regression using the default log link. The *P-*values between comparisons were adjusted using Holm’s test, which adjusts the calculation of probability in line with the number of comparisons made to avoid type I errors [45]. No offset was used since the same initial number of laboratory colony larvae (20) used to inoculate all samples. Negative binomial regression was utilized in lieu of logistic regression because negative binomial regression is better suited for data containing a large number of zero values.

Adult mosquito numbers were compared using piecewise negative binomial regression in SAS [46]. Separate slopes were fit for the before-treatment (12 June to 1 August) and after-treatment (5 August to 30 September) periods to examine whether the slope changed after placement of autodissemination stations in the treatment sites. We tested for the main effects of treatment, and of time in the before-treatment and time in the after-treatment periods, and for the interactions of treatment and time in both periods. These interactions were included to determine if the slopes differed between the treatment (HA1-3) and control (HC1-3) in either before or after-treatment periods. Time was treated as a continuous variable for the analysis of adult and egg data and as a categorical variable for the analysis of larval and pupal data.

## Results

### Egg populations

**Fig. 3:**
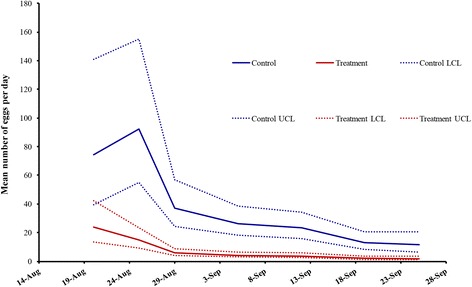
Least square means for treatment (*red line*) and control (*blue line*) sites with 95% confidence intervals for egg accumulation from oviposition cups and observed weekly mean egg counts by site. Five oviposition cups were deployed per hot spot each week for 7 weeks (one trap failure in the first week of trapping). Egg data were collected after the autodissemination stations were placed

Between 11 August and 29 September 2014, a total of 4770 eggs were collected from the six study sites. Fewer eggs were collected from oviposition cups in the treatment sites compared to those in the control sites throughout the course of the study (*χ* ^2^ = 42.56; *df* = 1, *P* < 0.0001; Fig. 3). The mean number of eggs collected in control sites were five times higher than the treatment sites, with 6.9 ± 10.1 (0–66) [mean ± standard deviation, (range)] per day per oviposition cup (Fig. 3). The mean number of eggs collected in treatment sites remained below two eggs per day per oviposition cup: 1.4 ± 2.7 (0–16).

### Larval populations and pupal mortality

The model used to analyze larval data showed that the study sites were significant predictor of the mean number of larvae present in the sentinel cups (*χ* ^2^ = 36.40, *df* = 5, *P* < 0.0001; Table 1). A linear contrast of the average number of larvae collected from HA1-3 (treatment sites) and from HC1-3 (control sites) showed a significantly lower mean number of larvae in the treatment sentinel cups (*Z* = -5.23, *P* < 0.0001; Table 1). Water samples collected from sentinel cups in the treatment sites resulted in higher pupal mortality of laboratory strain *Ae. albopictus* (12.4%) than did water samples from the control sites (0.58%) (*χ* ^2^ = 53.8, *df* = 1, *P* < 0.0001). LC-MS-MS analysis detected 0.00400 to 0.0113 ppb pyriproxyfen residue in water samples collected from the treatment sites. We observed 70% pupal mortality at 0.00400 ppb, and 100% at 0.00478 ppb, while the highest concentration of 0.0113 ppb achieved 95% pupal mortality.

**Table 1:** Number of larvae and dead pupae by site with least square means and 95% confidence intervals for treatments control

Site	No. cups	Larvae		Dead pupae	
		Mean	95% CI	Mean	95% CI
HA1	36	0.1	0–4.0	3.6	0–20.0
HA2	39	3.9	0–25.0	2.4	0–20.0
HA3	39	0.4	0–4.0	2.1	0–18.0
LS mean (95%CI)		0.5	0.3–0.9	2.2	0–0.9
HC1	40	1.8	0–19.0	0.1	0–2.0
HC2	36	4.1	0–38.0	0.2	0–2.0
HC3	39	7.4	0–50.0	0.1	0–2.0
LS mean (95% CI)		4.0	2.5–6.3	0.1	0.1–0.1

### *Aedes albopictus* adult populations

**Fig. 4:**
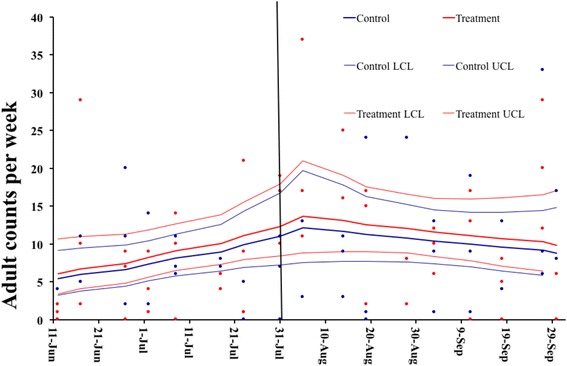
Least square means for treatment (*red line*) and control (*blue line*) sites with 95% confidence intervals and observed counts by site (treatment: *red circles*; control: *blue circles*) for total number of female and male adult *Ae. albopictus* collected in BGS traps. One BGS trap was deployed in each hot spot for 24 h once a week for 17 weeks. Autodissemination stations in the treatment sites on 28 July, 2014. *Black line* placed on 28 July indicates the oviposition cup, sentinel cup and autodissemination station deployment

A total of 623 adult mosquitoes were collected from treatment and control sites. The mean number of adults collected prior to autodissemination station deployment from control and treatment sites was 8.1 ± 8.7 (0–43) [mean ± SD, (range)] and 9.5 ± 9.2 (0–37) per day per BGS trap, respectively. Following the station deployment, the mean number of adults collected from control and treatment sites were 9.8 ± 8.7 (0–33) and 11.9 ± 11.8 (0–51) per day per BGS trap (Fig. 4). Neither interaction between treatment and time (before-and after-treatment) were significant, nor was the main treatment effect (*χ* ^2^ = 1.48, *df* = 1, *P* = 0.22; Fig. 4).

## Discussion

We assessed autodissemination as a means of reducing *Ae. albopictus* populations in hot spots, which was ultimately an attempt to manage this species area-wide. It has been shown that by targeting immature *Ae. albopictus* populations in hot spots, it is possible to achieve area-wide control [41]. Studies have demonstrated the efficacy of an autodissemination approach to reduce egg numbers in *Aedes* species in cages, rooms, and small scale field settings [10, 26, 32]. Our study targeted an area as large as 0.8 ha in an urban habitat and evaluated the impact of the stations on *Ae. albopictus* populations. We found egg and larval populations were reduced in autodissemination sites. These results were supported by higher pupal mortality during laboratory bioassays. There was no significant effect on the number of adult populations following autodissemination deployment; however, the overall trend in treatment and control sites were similar, indicating that adult numbers increased and decreased during repeated measurements of adult populations, mostly because our calculations were based on six traps (7 weeks). Even though we had long periods of follow-up with repeated measurements of adults using BGS traps to assess the effect of autodissemination station deployment, because of large variations in adult populations between sampling sites and across different sampling periods, the true effect on adults was not clear [47]. In addition, given the unique biology of container-inhabiting *Aedes* mosquitoes, we cannot discount recurring immigration into our sampling sites during the course of these studies. While the immigration of adults limited our ability to detect a decline in adult populations within the hot spots, these mosquitoes likely helped distribute pyriproxyfen to larval containers. This is supported by the detection of pyriproxyfen residue in treatment site sentinel cups, pupal mortality, and the presence of significantly less eggs and larvae compared to control site sentinel cups [33]. Although adult numbers were not significantly reduced, we recorded lower egg numbers from the treatment sites, which demonstrated that the stations were effectively contaminating adults and impacting their fecundity, leading to less egg collection from treatment sites. A previous study reported that pyriproxyfen contamination inhibits *Ae. albopictus* egg production [48]. In summary, adult movement makes it difficult to interpret the findings although we tried to reduce immigration by weekly ULV adulticiding events, however, measurements of all three immature stages support our main conclusion that autodissemination stations reduced *Ae. albopictus* populations in the sites. Our results on adult populations were inconclusive due to the low number of BGS traps deployed and immigration. The availability of additional adult data may still not capture the difference between treatment and control sites because adult immigration from adjacent sites was reduced but not eliminated by weekly adulticiding. Despite these limitations, the presence of less eggs and larvae could only be explained by sub-lethal exposure of the females to pyriproxyfen. For future studies, we will increase the number of treatment and control sites along with the number of BGS traps per site to improve adult surveillance. Barrier treatments using backpack and hand sprayers on vegetation in the buffer areas, will be applied as an alternative to ULV adulticiding to further decrease immigration into the study sites.

One of the strengths of the experimental design for this study was to investigate the effect of our treatment on each life stage of the mosquito. In addition to monitoring adult populations, we collected larvae and water from sentinel cups for bioassays. The water was used to challenge laboratory colonies which allowed us to determine the effects of pyriproxyfen on pupal stages. We also used residue data to confirm whether or not pyriproxyfen is disseminating in the field. Correlation between the mortality in field collected cups and the residue analysis of samples is challenging as the amount of organic content varies in field collected samples. The organic content degrades and adsorbs pyriproxyfen, which makes it harder to extract from the water phasic substrates using organic solvents. Most of the time, this process resulted in loss of pyriproxyfen and that produced variations in the results [49]. Whereas in the larval bioassay, larvae are continuously exposed to the same water for 2–3 weeks, and organic contents degrade over time, which provides regular supply as similar to slow release granule formulations of insecticides.

The autodissemination approach presented here shows promise to be a new tool for the control of a mosquito responsible for reducing quality of life and initiating disease outbreaks. *Aedes albopictus* is difficult to control and integrated abatement methods must be used to suppress the populations [15, 17, 19, 22, 37, 50]. Autodissemination station deployments can be incorporated with existing integrated mosquito management programs to increase the effectiveness while reducing time, cost, and effort spent for methods such as door-to-door source reduction. Target containers (plant pot saucers, recycle bins, used tires, etc.) are usually located within private residential parcels which are sometimes [51] inaccessible to mosquito control personnel. Even if inspectors can gain access to the parcels and treat all containers with larvicides, new ones frequently appear [13]. This approach is labor- and cost-intensive, while the present study indicates that this exceptionally low risk and relatively inexpensive ($ 4.5 to $ 100 per kg) active ingredient, pyriproxyfen, offers promise. Pyriproxyfen is certainly deserving of further investigations, with the possible outcome of a modest label modification.

Another challenge with *Ae. albopictus* is its use of cryptic habitats for larval development. Unlu et al. [37] reported a dominant representation of *Ae. albopictus* in corrugated extension spouts [16]. Because container-inhabiting *Aedes* species prefer cryptic larval habitats, which are difficult to reach by traditional mosquito control techniques [9, 52], the autodissemination approach may be a uniquely useful tool because it exploits female oviposition behavior to deliver a toxicant to these habitats [53]. Container-inhabiting *Aedes* species also exhibit skip-oviposition and oviposit in a wide number and variety of artificial containers within peridomestic habitats [13, 53]. The autodissemination approach provides a unique opportunity for vector control operations to utilize skip-oviposition to their advantage globally.

## Conclusions

Despite ongoing advances in the biology and ecology of *Ae. albopictus* and other container-inhabiting mosquito species, effective vector control remains as the primary option for the protection of human health and comfort. *Aedes albopictus* is also poised to expand its range in the next few decades; primarily because of warmer winter temperatures [54]. The lack of effective area-wide control measures will present a major challenge to mosquito control agencies tasked with the protection of public health. Better planning and integration of new control products and methods, such as the autodissemination approach, will be the key for successful intervention campaigns.

## Additional file

Additional file 1: Data generated and analysed in this study. (XLSX 23 kb)

## Abbreviations

BGS traps: BG sentinel traps
Bti: *Bacillus thuringienis* var. *israelensis*
CHIKV: Chikungunya virus
LC-MS-MS: Liquid-mass-mass spectrophotometry
ULV: Ultra-low volume
ZIKV: Zika virus

## References

[1] CDC CfdcaP (2016) Chikungunya in the Caribbean. https://wwwnc.cdc.gov/travel/notices/watch/chikungunya-caribbean. Accessed 1 Feb 2017.

[2] Delatte H, Dehecq JS, Thiria J, Domerg C, Paupy C, Fontenille D (2008). Geographic distribution and developmental sites of *Aedes albopictus* (Diptera: Culicidae) during a Chikungunya epidemic event. Vector Borne Zoonotic Dis.

[3] LaDeau SL, Allan BF, Leisnham PT, Levy MZ (2015). The ecological foundations of transmission potential and vector-borne disease in urban landscapes. Funct Ecol.

[4] Campos GS, Bandeira AC, Sardi SI (2015). Zika virus outbreak, Bahia, Brazil. Emerging Infect Dis.

[5] Ruiz-Moreno D, Vargas IS, Olson KE, Harrington LC (2012). Modeling dynamic introduction of Chikungunya virus in the United States. PLoS Negl Trop Dis.

[6] Schuffenecker I, Iteman I, Michault A, Murri S, Frangeul L, Vaney MC, et al. Genome microevolution of chikungunya viruses causing the Indian Ocean outbreak. PLoS Med.2006;3:e263. 10.1371/journal.pmed.0030263 PMC1463904 16700631

[7] Tsetsarkin KA, Vanlandingham DL, McGee CE, Higgs S (2007). A single mutation in chikungunya virus affects vector specificity and epidemic potential. PLoS Pathog.

[8] Preechaporn W, Jaroensutasinee M, Jaroensutasinee K (2006). The larval ecology of *Aedes aegypti* and *Ae. albopictus* in three topographical areas of Southern Thailand. Dengue Bull.

[9] Unlu I, Farajollahi A, Strickman D, Fonseca DM (2013). Crouching tiger, hidden trouble: Urban sources of *Aedes albopictus* (Diptera: Culicidae) refractory to source-reduction. PLoS One.

[10] Caputo B, Ienco A, Cianci D, Pombi M, Petrarca V, Baseggio A, et al. Auto-dissemination approach: a novel concept to fight *Aedes albopictus* in urban areas. PLoS Negl Trop Dis. 2012;6:e1793. 10.1371/journal.pntd.0001793 PMC3429402 22953015

[11] Bartlett-Healy K, Unlu I, Obenauer P, Hughes T, Healy S, Crepeau T, et al. Larval mosquito habitat utilization and community dynamics of *Aedes albopictus* and *Aedes japonicus* (Diptera: Culicidae). J Med Entomol. 2012;49:813–24. 10.1603/me11031 22897041

[12] Chan K, Ho B, Chan Y (1971). *Aedes aegypti* (L.) and *Aedes albopictus* (Skuse) in Singapore City: 2. Larval habitats*. Bull WHO.

[13] Richards SL, Apperson CS, Ghosh SK, Cheshire HM, Zeichner BC (2006). Spatial analysis of *Aedes albopictus* (Diptera: Culicidae) oviposition in suburban neighborhoods of a Piedmont community in North Carolina. J Med Entomol.

[14] Fonseca D, Kaplan L, Heiry R, Strickman D (2015). Density-dependent oviposition by female *Aedes albopictus* (Diptera: Culicidae) spreads eggs among containers during the summer but accumulates them in the fall. J Med Entomol.

[15] Richards SL, Ghosh SK, Zeichner BC, Apperson CS (2008). Impact of source reduction on the spatial distribution of larvae and pupae of *Aedes albopictus* (Diptera: Culicidae) in suburban neighborhoods of a Piedmont community in North Carolina. J Med Entomol.

[16] Unlu I, Faraji A, Indelicato N, Fonseca DM (2014). The hidden world of Asian tiger mosquitoes: immature *Aedes albopictus* (Skuse) dominate in rainwater corrugated extension spouts. Trans R Soc Trop Med Hyg.

[17] Wheeler AS, Petrie WD, Malone D, Allen F (2009). Introduction, control and spread of *Aedes albopictus* on Grand Cayman Island, 1997-2001. J Am Mosq Control Assoc.

[18] Estrada-Franco JG, Craig GB. Biology, disease relationships, and control of *Aedes albopictus*. Pan American Health Organization Pan American Sanitary Bureau, Regional Office of the World Health Organization. 1995.

[19] Sun D, Williges E, Unlu I, Healy S, Williams GM, Obenauer P, et al. Taming a tiger in the city: comparison of motorized backpack applications and source reduction against the Asian tiger mosquito, *Aedes albopictus*. J Am Mosq Control Assoc. 2014;30:99–105. 10.2987/13-6394.1 25102592

[20] Williams GM, Faraji A, Unlu I, Healy SP, Farooq M, Gaugler R, et al. Area-wide ground applications of *Bacillus thuringiensis* var. *israelensis* for the control of *Aedes albopictus* in residential neighborhoods: from optimization to operation. PLoS One. 2014;9:e110035. 10.1371/journal.pone.0110035 PMC4203744 25329314

[21] Zhou YB, Zhao TY, Leng PE (2009). Evaluation on the control efficacy of source reduction to *Aedes albopictus* in Shanghai, China. Chin J Vec Biol Contr.

[22] Fonseca DM, Unlu I, Crepeau T, Farajollahi A, Healy SP, Bartlett‐Healy K, et al. Area-wide management of *Aedes albopictus*. Part 2: gauging the efficacy of traditional integrated pest control measures against urban container mosquitoes. Pest Manage Sci. 2013;69:1351–61. 10.1002/ps.3511 23649950

[23] Bartlett-Healy K, Hamilton G, Healy S, Crepeau T, Unlu I, Farajollahi A, et al. Source reduction behavior as an independent measurement of the impact of a public health education campaign in an integrated vector management program for the Asian tiger mosquito. Int J Env Res Public Health. 2011;8:1358–67. 10.3390/ijerph8051358 PMC3108114 21655124

[24] Gaugler R, Suman D, Wang Y (2012). An autodissemination station for the transfer of an insect growth regulator to mosquito oviposition sites. Med Vet Entomol.

[25] Wang Y, Suman DS, Bertrand J, Dong L, Gaugler R (2014). Dual treatment autodissemination station with enhanced transfer of an insect growth regulator to mosquito oviposition sites. Pest Manage Sci.

[26] Mains JW, Brelsfoard CL, Dobson SL (2015). Male mosquitoes as vehicles for insecticide. PLoS Negl Trop Dis.

[27] Geden CJ, Devine GJ (2012). Pyriproxyfen and house flies (Diptera: Muscidae): effects of direct exposure and autodissemination to larval habitats. J Med Entomol.

[28] Snetselaar J, Andriessen R, Suer RA, Osinga AJ, Knols B, Farenhorst M (2014). Development and evaluation of a novel contamination device that targets multiple life-stages of *Aedes aegypti*. Parasit Vectors.

[29] Kawada H, Shono Y, Ito T, Abe Y (1993). Laboratory evaluation of insect growth regulators against several species of anopheline mosquitoes. Jap J Sanit Zool.

[30] Itoh T (1995). Utilization of blood fed females of *Aedes aegypti* as a vehicle for the transfer of the insect growth regulator, pyriproxyfen, to larval habitats. Tropical Medicine.

[31] Trexler JD, Apperson CS, Schal C (1998). Laboratory and field evaluations of oviposition responses of *Aedes albopictus* and *Aedes triseriatus* (Diptera: Culicidae) to oak leaf infusions. J Med Entomol.

[32] Devine GJ, Perea EZ, Killeen GF, Stancil JD, Clark SJ, Morrison AC (2009). Using adult mosquitoes to transfer insecticides to *Aedes aegypti* larval habitats. Proc Natl Acad Sci U S A.

[33] Abad-Franch F, Zamora-Perea E, Ferraz G, Padilla-Torres SD, Luz SL (2015). Mosquito-disseminated pyriproxyfen yields igh breeding-site coverage and boosts juvenile mosquito mortality at the neighborhood scale. PLoS Negl Trop Dis.

[34] Dash AP, Ranjit MR (1992). Comparative efficacy of aphid extracts and some juvenoids against the development of mosquitoes. J Am Mosq Control Assoc.

[35] Ponlawat A, Fansiri T, Kurusarttra S, Pongsiri A, McCardle PW, Evans BP, Richardson JH (2013). Development and evaluation of a pyriproxyfen-treated device to control the dengue vector, *Aedes aegypti* (L.) (Diptera:Culicidae). Southeast Asian J Trop Med Public Health.

[36] Unlu I, Farajollahi A, Healy SP, Crepeau T, Bartlett‐Healy K, Williges E, et al. Area-wide management of *Aedes albopictus*: choice of study sites based on geospatial characteristics, socioeconomic factors and mosquito populations. Pest Manage Sci.2011;67:965–74. 10.1002/ps.2140 21452166

[37] Unlu I, Farajollahi A, Ilia R, Crepeau TN, Daniel S, Gaugler R (2014). Differences in male-female ratios of *Aedes albopictus* (Diptera: Culicidae) following ultra-low volume adulticide applications. Acta Trop.

[38] Carrieri M, Bellini R, Maccaferri S, Gallo L, Maini S, Celli G (2008). Tolerance thresholds for *Aedes albopictus* and *Aedes caspius* in Italian urban areas. J Am Mosq Control Assoc.

[39] Read NR, Rooker JR, Gathman JP (1994). Public perception of mosquito annoyance measured by a survey and simultaneous mosquito sampling. J Am Mosq Control Assoc.

[40] Kroeckel U, Rose A, Eiras ÁE, Geier M (2006). New tools for surveillance of adult yellow fever mosquitoes: comparison of trap catches with human landing rates in an urban environment. J Am Mosq Control Assoc.

[41] Unlu I, Klingler K, Indelicato N, Faraji A, Strickman D (2015). Suppression of *Aedes albopictus*, the Asian tiger mosquito, using a “hot spot” approach. Pest Manage Sci.

[42] Farajollahi A, Crans SC (2012). A checklist of the mosquitoes of New Jersey with notes on established invasive species. J Am Mosq Control Assoc.

[43] Stojanovich CJ. Illustrated key to common mosquitoes of northeastern North America. Cullom & Ghertner Nashville. 1961.

[44] Trexler JD, Apperson CS, Zurek L, Gemeno C, Schal C, Kaufman M, et al. Role of bacteria in mediating the oviposition responses of *Aedes albopictus* (Diptera: Culicidae). J Med Entomol. 2003;40:841–8. 10.1603/0022-2585-40.6.841 14765661

[45] Holm S. A simple sequentially rejective multiple test procedure. Scand J Stat. 1979;65–70.

[46] Marsh LC, Cormier DR. Spline Regression Models, Quantitative Applications in the Social Sciences No. 137. Thousand Oaks: Sage; 2002.

[47] Wilson AL, Boelaert M, Kleinschmidt I, Pinder M, Scott TW, Tusting LS, Lindsay SW (2015). Evidence-based vector control? Improving the quality of vector control trials. Trends Parasito.

[48] Ohba SY, Ohashi K, Pujiyati E, Higa Y, Kawada H, Mito N, Takagi M (2013). The effect of pyriproxyfen as a “population growth regulator” against *Aedes albopictus* under semi-field conditions. PLoS One.

[49] Picó Y, Fernández M, Ruiz MJ, Font G (2007). Current trends in solid-phase-based extraction techniques for the determination of pesticides in food and environment. J Biochem Biophys Methods.

[50] Farajollahi A, Healy SP, Unlu I, Gaugler R, Fonseca DM (2012). Effectiveness of ultra-low volume nighttime applications of an adulticide against diurnal *Aedes albopictus*, a critical vector of dengue and chikungunya viruses. PLoS One.

[51] Hawley WA (1998). The biology of *Aedes albopictus*. J Am Mosq Control Assoc.

[52] Benedict MQ, Levine RS, Hawley WA, Lounibos LP (2007). Spread of the tiger: global risk of invasion by the mosquito *Aedes albopictus*. Vector Borne Zoonotic Dis.

[53] Davis TJ, Kaufman PE, Tatem AJ, Hogsette JA, Kline DL. Development and evaluation of an attractive self-marking ovitrap to measure dispersal and determine skip oviposition in *Aedes albopictus* (Diptera: Culicidae) field populations. J Med Entomol. 2016;53(1):31-8. 10.1093/jme/tjv170 PMC4723682 26534725

[54] Rochlin I, Ninivaggi DV, Hutchinson ML, Farajollahi A (2013). Climate change and range expansion of the Asian tiger mosquito (*Aedes albopictus*) in Northeastern USA: implications for public health practitioners. PLoS One.

